# Crimean-Congo hemorrhagic fever in pregnancy: A systematic review and case series from Russia, Kazakhstan and Turkey

**DOI:** 10.1016/j.ijid.2017.02.019

**Published:** 2017-05

**Authors:** Natalia Yurievna Pshenichnaya, Hakan Leblebicioglu, Ilkay Bozkurt, Irina Viktorovna Sannikova, Gulzhan Narkenovna Abuova, Andrey Sergeevich Zhuravlev, Sener Barut, Mutabar Bekovna Shermetova, Tom E. Fletcher

**Affiliations:** a Rostov State Medical University, Department of Infectious Diseases, Rostov-on-Don, Russia; b Ondokuz Mayis University, Department of Infectious Diseases and Clinical Microbiology, Samsun, Turkey; c Stavrapol State Medical University, Department of Infectious Diseases and Tuberculosis, Stavropol, Russia; d South-Kazakhstan State Pharmaceutical Academy, Department of Infectious Diseases and Dermatovenerology, Shymkent, Kazakhstan; e First State Medical University «I. M. Sechenov», Research Department, Moscow, Russia; f Gaziosmanpasa University, Tokat, Turkey; g Turkestan Central City Hospital, Department of Infectious Diseases,Turkestan, Kazakhstan; h Liverpool School of Tropical Medicine, Liverpool, United Kingdom

**Keywords:** Crimean-Congo hemorrhagic fever, pregnancy, healthcare associated infection, viral hemorrhagic fever

## Abstract

**Background:**

Crimean-Congo hemorrhagic fever (CCHF) is acute viral infection and a major emerging infectious diseases threat, affecting a large geographical area. There is no proven antiviral therapy and it has a case fatality rate of 4–30%. The natural history of disease and outcomes of CCHF in pregnant women is poorly understood.

**Objectives:**

To systematically review the characteristics of CCHF in pregnancy, and report a case series of 8 CCHF cases in pregnant women from Russia, Kazakhstan and Turkey.

**Methods:**

A systematic review was performed following the Preferred Reporting Items for Systematic Reviews and Meta-Analyses (PRISMA) statement protocol. PubMed, SCOPUS, Science Citation Index (SCI) were searched for reports published between January 1960 and June 2016. Two independent reviewers selected and reviewed studies and extracted data.

**Results:**

Thirty-four cases of CCHF in pregnancy were identified, and combined with the case series data, 42 cases were analyzed. The majority of cases originated in Turkey (14), Iran (10) and Russia (6). There was a maternal mortality of 14/41(34%) and fetal/neonatal mortality of in 24/41 cases (58.5%). Hemorrhage was associated with maternal (p = 0.009) and fetal/neonatal death (p < 0.0001). There was nosocomial transmission to 38 cases from 6/37 index pregnant cases.

**Conclusion:**

Cases of CCHF in pregnancy are rare, but associated with high rates of maternal and fetal mortality, and nosocomial transmission.

## Highlights

• CCHF in pregnancy is rare but has high rates of maternal (34%) and fetal mortality (59%).
• Maternal hemorrhage is associated with maternal and fetal/neonatal death.
• Nosocomial transmission of CCHF from 6/37 index pregnant cases resulted in 38 cases.
• Early recognition and risk-assessment allows appropriate IP & C precautions and supportive care provision.

## Introduction

Crimean-Congo hemorrhagic fever (CCHF) is an acute tick-borne viral infection and a major emerging infectious diseases threat. It affects a wide geographical area, centered in Eurasia including Turkey, Russia and Kazakhstan but is under-reported and diagnosis is often delayed. Fever, thrombocytopenia and hemorrhage are the characteristic clinical features, with supportive care forming the mainstay of treatment protocols, although ribavirin is utilized by some centers. Provision of blood product support and access to critical care interventions can improve outcomes, with reported case fatality rates (CFR) being 4–20%.^1^

The majority of cases of CCHF report a history of tick bite, but healthcare related transmission of CCHF is well reported, and occurs in both high and low-resource settings. Failure to recognize CCHF and as a result implement appropriate infection, prevention and control procedures results in significant nosocomial risk, especially in the context of critical care interventions.^2^, ^3^, ^4^ Retrospective analysis from Turkey has demonstrated that needle stick injuries are the most frequent cause of high risk exposures, followed by ‘splash’ exposures to mucous membranes and highlighted a potential benefit of ribavirin post-exposure prophylaxis.^5^

Although the immune system changes in pregnancy are not completely understood, pregnant woman may be more likely to acquire certain infectious diseases such as toxoplasmosis, and be more severely affected by others such as influenza and varicella.^6^ Viral hemorrhagic fevers such as Ebola virus disease and Lassa Fever are more severe in pregnancy,^7^, ^8^ and frequently result in spontaneous abortion with additional nosocomial risk. Although clinical and epidemiological CCHF data are increasingly reported, few data exist on CCHF in pregnancy.^9^, ^10^, ^11^ The mortality of CCHF disease in pregnant women appears to be higher than in the general population (up to 33%),^10^ and the severe course of CCHF in pregnant women may also increase risk of nosocomial infection in health care settings.^2^

In this study we aimed to systematically review the characteristics of CCHF cases in pregnancy, and to report an additional case series of 8 CCHF cases in pregnant women from Russia, Kazakhstan and Turkey.

## Material and methods

We planned and reported this systematic review in accordance with guidelines for performing and reporting systematic reviews and meta-analyses (PRISMA, Preferred Reporting Items for Systematic Reviews and Meta-Analyses). We searched PubMED, Science Citation Index (SCI) and Scopus databases for English and foreign language studies published between January 1960 and June 2016. The keyword ‘Crimean Congo H(a)hemorrhagic Fever” was utilised then combined with “pregnancy”; ‘pregnant’ and ‘vertical’. We also searched reference sections; Google scholar and reviews for other studies. Statistical analyses were performed with the use of IBM SPSS Statistics for Windows; Version 24.0. Armonk; NY: IBM Corp.

## Study Selection

Two reviewers (HL & IB) independently screened the titles and abstracts of all studies that were identified through database searches. Inclusion criteria were (1): report of a case of laboratory confirmed Crimean-Congo Hemorrhagic fever OR report of a clinical case with a direct epidemiological link to a laboratory confirmed case of Crimean-Congo hemorrhagic fever and (2); pregnancy. Full-text copies of potentially relevant studies were retrieved and reviewed independently (TF & IB), extracting data from each study meeting inclusion criteria. Standardised data was extracted from each case when available including date, age, gestation, laboratory diagnosis, outcome of mother and foetus, hemorrhagic manifestations and secondary cases.

## Results

**Figure 1:**
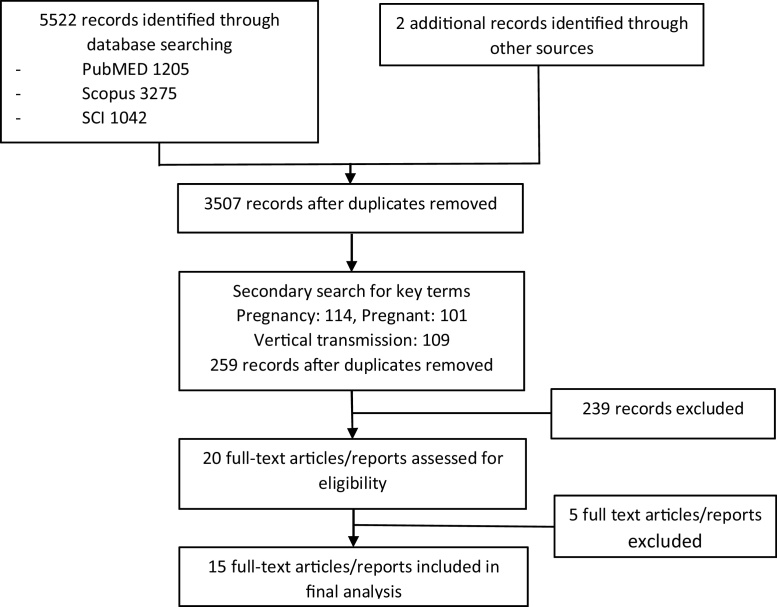
Flowchart of literature search.

The initial search results identified the following number of records: Scopus 3275; PubMED 1205; and SCI 1042. After removal of duplicates 3507 records were combined with secondary search terms (Pregnancy: 101, pregnant:114, vertical:109,). An additional search by Google scholar identified 2 further reports. 20 full text articles were then retrieved, with the total number of 34 CCHF cases identified in pregnancy in 15 articles (Figure 1). An additional 8 cases were added from this case series from Russia (5 cases), Kazakhstan (2 cases) and Turkey (1 case), resulting in total of 42 cases of CCHF in pregnancy (Table 1).

The first report was from a case in 1979 and the last case occurred in 2016, with cases from Turkey (14 cases), Iran (10 cases), Russia (6 cases), former Yugoslavia (4 cases), Iraq (3 cases), Kazakhstan (2 cases), Mauritania (2 cases) and Bulgaria (1 case). Gestation was reported in 29 cases, with 11 cases occurring in the first 20 weeks of pregnancy and 18 cases occurring in the second 20 weeks of pregnancy.

Maternal and fetal/neonatal outcome was reported in 41 cases, with 14/41 cases (34%) reporting maternal death, and foetal/neonatal death occurring in 24/41 cases (58.5%). In the first 20 weeks of pregnancy there were 1/11 maternal deaths and 6/11 fetal deaths, and in weeks 20–40 of pregnancy there were 8/18 maternal deaths and 9/18 fetal/neonatal deaths. There was no statistically significant difference between maternal deaths in the 1st 20 weeks of pregnancy compared to weeks 20–40 (p = 0.096–Fisher’s exact test, two-tailed). There was no haemorrhage in 10/41 cases, with maternal and fetal survival occurring in all 10 cases. When maternal haemorrhage was reported (vaginal or other sites), there were 14/31 maternal deaths and 24/31 fetal/neonatal deaths. When compared to the non-haemorrhage group (0/10) differences in outcome were statistically significant (p = 0.009, and p < 0.0001 respectively, Fisher’s exact test, two-tailed). Nosocomial transmission from pregnant CCHF cases was reported in 6/37 cases, resulting in additional 38 cases.

## CCHF case series

### Case 1 (Stavropol region, Russia, 2002)

A 20-year-old pregnant woman (16-weeks gestation) was admitted to the infectious diseases hospital on the 3rd day of disease with fever (38,5 °C), headache, loss of appetite and a petechial rash on her lower limbs. She was an agriculture worker and had contact with animals, but denied any history of tick bite. Initial laboratory tests were: Hb 87 g/l, RBC 3.2 × 10^12^/L, WBC 3.4 × 10^9^/L, PLT 112 × 10^9^/L, APTT 46 secs. CCHF was suspected and confirmed by PCR on the 4th day of disease, with a positive ELISA result in the second week. Empirical antibiotics and supportive treatment were given (no Ribavirin) and 2 days later her condition improved, and temperature normalized. Laboratory tests improved (WBC 7.5 × 10^9^/L, PLT 144 × 10^9^/L) and she was discharged after a 16-day admission. At 38 weeks of pregnancy she gave birth to a healthy child.

### Case 2 (Stavropol region, Russia, 2003)

A 19-year-old pregnant woman (38-weeks gestation) was admitted to the infectious diseases hospital with a one-day history of fever (up to 39^ °^C), headache, and myalgia. She lived in a rural area and several days previously reported removing ticks from her cow. CCHF was suspected and laboratory tests at admission were: Hb 95 g/L, RBC 3.2 × 10^12^/L, WBC 3.8 × 10^9^/L, PLT 132 × 10^9^/L and APTT 46 secs. Ribavirin, empiric antimicrobials and supportive treatment were commenced and 2–3 days later her condition improved, and her fever settled. Her laboratory tests improved by day 8 (HB 110 g/l, RBC 3.8 × 10^12^/L, WBC 4.2 × 10^9^/L, PLT 153 × 10^9^/L, APTT 44 secs) and she had no hemorrhagic manifestations. CCHF was confirmed by ELISA and on day 11 she was transferred to a maternity unit, where 3 days later she gave birth to a healthy baby.

### Case 3 (Izobilnensky district, Stavropol region, Russia, 2004)

A 17-year-old pregnant woman (30-weeks gestation) was admitted to the gynecology department with a 2-day history of high fever (>39 °C) and back pain. She lived in rural area (with suspected tick contact) and laboratory tests were: HB 102 g/L, RBC 3.5 × 10^12^/L, WBC 5.6 × 10^9^/L, PLT 153 × 10^9^/L, ESR 20 mm/h, APTT 43 secs, leukocyturia and proteinuria (2 g/l). Acute pyelonephritis was the initial diagnosis and anti-bacterial and supportive treatment was started, but did not result in any significant improvement in her clinical condition.

On the 4th day of illness a hemorrhagic rash appeared on her lower limbs, with ecchymosis developing on the skin of her chest and abdomen. She became hypotensive (90/60 mmHg), with increased respiratory rate (24/min), and worsening of her laboratory parameters (Hb72 g/L, RBC 2.1 × 10^12^/L, WBC 5.6 × 10^9^/L, Plt 32 × 10^9^/L and APTT 48 secs). CCHF was suspected and confirmed by PCR (CCHF IgM negative) and she was transferred to the infectious diseases department. On the 5th day of illness there was onset of premature labour and a stillborn baby was delivered. This was further complicated by post-partum and gastrointestinal tract hemorrhage, development of acute respiratory distress syndrome and the patient died on the 6th day of disease. Ribavirin was not administered to the patient.

### Case 4 (Malgobeksky district, Republic of Ingushetia, Russia, 2005)

A 24-year-old pregnant physician (17-weeks gestation) presented with a 4-day history of fever (39 °C), thirst, abdominal pain, vomiting and cough. She had had previous occupational exposure during phlebotomy and processing of blood samples from a 75-year old patient (index patient), who died of massive gastrointestinal hemorrhage.^12^ Two daughters, who provided care at home also died. The pregnant physician was initially hospitalized in the gynecology department and on the 10th day of illness was transferred to a regional infectious department with suspected viral hepatitis. At this stage she had developed jaundice, ecchymosis at injection sites, a hemorrhagic rash, hepatomegaly, splenomegaly and a right-sided pneumonia. She was managed with antimicrobial therapy and supportive treatment, and was transferred to the National Scientific Research Institute of obstetrics and pediatrics in Moscow (NSRIOP) on the 12th day of disease. She remained febrile, and became critically unwell with respiratory distress and was transferred to the Intensive care unit (ICU) (Hb 60 g/L, PLT 400 × 10^9^/L, WBC 10.7 × 10^9^/L, ESR 40 mm/h, APTT 58 secs).

She was subsequently transferred to the ICU of National Institute of hematology in a comatose condition with a suspected hematological disease, but following infectious diseases review, CCHF was suspected and she was transferred to the ICU of infectious diseases hospital on the 16 day of disease. Progressive pneumonia, myocarditis, hepatic insufficiency, antenatal fetal death, and a disseminated intravascular coagulation (DIC)-syndrome were diagnosed at this point. A caesarean section was performed on the 18th day of illness and a dead fetus without hemorrhagic manifestations was extracted. The patient remained on a ventilator for several weeks, and after her condition improved she was extubated and then discharged from hospital 2 months later. CCHF RT-PCR from samples taken on day 15 were negative, but the diagnosis confirmed by ELISA with positive CCHF IgM and increasing titers of IgG. The patient was not treated with ribavirin.

### Case 5 (Turkestan city, Southern-Kazakhstan region, Kazakhstan, 2009)

A 23-year-old woman was re-admitted to a maternity unit with her 7-day-old newborn 3 days after discharge, with high fever and vaginal bleeding. Post-partum endometritis with secondary post-partum hemorrhage was suspected, and emergency hysterectomy was performed for severe blood loss. Intra-abdominal hemorrhage continued and despite two further laparotomies she died. A few days later her baby also died. CCHF was diagnosed in the woman based on post-mortem results, and it was suspected that she acquired this in the last week of pregnancy. Two clinicians who performed surgical interventions on patient, and the pediatrician who managed the newborn (without direct contact with the mother) also contracted CCHF and died. Diagnosis was confirmed was confirmed by immuno-histochemical analysis of samples at post-mortem. Two more HCWs who cared for woman also developed CCHF, but survived.

### Case 6 (Turkestan city, Southern-Kazakhstan region, Kazakhstan, 2010)

A 21-year-old pregnant woman (34-weeks gestation) was admitted to the maternity hospital with a 4-day history high fever (>39 °C), dizziness, thirst and anorexia. She also complained of left sided abdominal/flank pain, was hypotensive (BP 90/60) and no fetal heart beat was found. She lived in rural area with animal contact, but denied a history of tick bite. The initial diagnosis was acute pyelonephritis and antenatal death, but CCHF was then suspected due to the thrombocytopenia (PLT 23 × 10^9^/L) in her initial blood tests. Hematomas were also observed at injection sites, and she then deteriorated rapidly with loss of consciousness, upper gastrointestinal hemorrhage, and uterine bleeding, dying 7 hours after admission. CCHF viral antigen was subsequently detected by immuno-histochemical staining of post-mortem tissues samples. One health care worker (nurse) who managed the patient wearing only gloves as personal protective equipment, later developed confirmed CCHF.

### Case 7 (Rostov-on-Don, Russia, 2011)

A 17-year-old pregnant woman (18-weeks gestation) was admitted to infectious diseases hospital with a 2-day history of fever (up to 39 °C), headache, and myalgia. Her initial laboratory tests were: Hb 90 g/L, RBC 3.4 × 10^12^/L, WBC 3.2 × 10^9^/L, PLT 162 × 10^9^/L and APTT 44 secs. She had a history of tick bite 7 days previously, CCHF was suspected and confirmed by RT-PCR on day 3 of disease. Ribavirin, antimicrobial and supportive treatment were started and 2–3 days later her condition improved, temperature decreased and laboratory results improved (WBC 8,7 × 10^9^/L, PLT 232 × 10^9^/L and APTT 43 secs). Hemorrhage was not evident during her disease course and she was discharged from hospital after 10 days and at term gave birth to a healthy child.

### Case 8 (Tokat, Turkey, 2016)

A 20-year-old woman in early pregnancy (estimated 4-weeks gestation) was admitted to Tokat State Hospital 1 day after a tick bite with a history of fever, sore throat, back pain anorexia, nausea and vomiting. Laboratory tests showed: WBC 1.75 × 10^9^/L, Plt 46 × 10^9^/L, APTT 53 secs, PT 17.2, AST 233 IU/L and ALT 98 IU/L. CCHF was suspected and RT-PCR positive from day 2 of illness. On day 3 of illness she developed vaginal bleeding and was referred to Tokat University Hospital with progressive thrombocytopenia (WBC 1.65 × 10^9^/L, Plt 23 × 10^9^/L, APTT 53 sec, PT 17.2, AST 270 IU/L, ALT 127 IU/L). She received 2 units of fresh frozen plasma and 1 unit of platelets and ribavirin was not given. Pelvic ultrasound showed a thickened endometrium with minimal cervical bleeding on examination. Her Beta HCG progressively reduced (2500IU − 143IU) and a complete spontaneous abortion was confirmed. She clinically improved and was discharged 9 days after admission.

## Discussion

Crimean-Congo hemorrhagic fever has been designated a priority global diseases threat by the World Health Organization.^12^ Its pathophysiology remains incompletely understood, particularly in vulnerable groups, despite recent increases in research efforts.^13^ Other viral hemorrhagic fevers, such as Ebola virus and Lassa fever, are thought to be more severe in pregnancy, resulting in higher maternal mortality rates. Pregnant patients with VHF also present additional infection, prevention and control challenges, due to the interventions required for obstetric complications and potential viral persistence in the fetus/products of conception. Limited data exists describing the course of CCHF in pregnancy, and we aimed to improve knowledge in this area through a systematic review of published cases and multi-national case series.

The most complete data set previously reported was by Gozel et al. in 2014 who published a case series of 5 pregnant women with CCHF and summary data on 21 other reported cases.^10^ Our systematic review identified an additional 7 reports (10 cases), that combined with our large additional case series provided a total 42 cases of CCHF in pregnancy for analysis. CCHF appears to be associated with more severe disease in pregnancy, with 14/41 cases (34%) resulting in maternal death. This is higher than overall case fatality rates in Turkey (5%),^14^ Russia (4%)^15^ and Kazakhstan (14.8%),^16^ but may reflect reporting bias, and national sub-group analysis of pregnant cases shows rates to be more comparable (pregnant cases from Turkey 1/14, 9.3%). There is however, a high rate of fetal/neonatal loss occurring in 24/41 cases (58.5%). In the majority of cases this is through spontaneous abortion early in pregnancy or associated with maternal death. Still birth appears to be rare as does neonatal death.

The stage of the pregnancy when CCHF occurs may also influence maternal mortality, with 8/18 mothers dying in the second half of pregnancy (>20 weeks) compared with 1/11 dying in the first half of pregnancy (<20 weeks), although this was not statistically significant (p = 0.096). In our cases series 3/8 cases resulted in maternal death occurring at 34, 38 and 40-weeks gestation. However, as we have highlighted in cases 4 & 8, with appropriate critical care interventions and blood product support, positive outcomes are possible in the context of severe CCHF in pregnancy. This was also demonstrated by Ergonul et al.,^9^ who reported a case of severe CCHF in late pregnancy that survived, but required 22 units of fresh frozen plasma, 54 units of platelets and repeat surgical intervention for ongoing uterine hemorrhage after caesarian section.

Due to the risk of nosocomial transmission, surgical interventions in pregnant women with CCHF require careful infection prevention and control planning and risk assessment. Although caesarian section was performed in 2 cases that we have summarized, with no secondary nosocomial transmission, the patients had tested RT-PCR negative in blood prior to the operations. The PCR status of the fetus and amniotic fluid at the time surgery was not known, but in the case reported by Ergonul et al., the neonate subsequently tested CCHF RT-PCR positive and died.^9^ However, surgery was undertaken for post-partum hemorrhage in case 5 resulting in nosocomial transmission to 2 surgeons, and the neonate also transmitted CCHF to a further 3 healthcare workers. The additional nosocomial risk of pregnancy in other VHFs is not well understood, and during the Ebola outbreak in West Africa there were significant limitations on pregnant women’s access to obstetric care. However, complicated cases were managed successfully,^17^ and caesarian sections were undertaken by one group in Monrovia with no associated nosocomial transmission (Dr J Brown personal communication).

Six of the 42 pregnant CCHF cases we identified did result in subsequent cases of CCHF as a result of nosocomial transmission. Naderi et al.,^18^ reported a nosocomial outbreak where a pregnant woman was an index case that infected another pregnant woman whilst sharing a room, who then infected another 4 healthcare workers. Nabeth et al.,^19^ conducted an investigation into a large nosocomial outbreak in Mauritania in 2003, identifying a 30-year old pregnant woman with severe CCHF as the index case. She directly infected a total of 15 healthcare workers, patients and visitors in the ward and emergency room setting with six fatal cases. A more recent report from Russia^2^ also highlighted the nosocomial risk of critical care interventions, particularly aerosol generating procedures that resulted in 8 healthcare worker infections, from a 23-year old pregnant woman. In all these cases CCHF was not initially suspected. The initial presentation of CCHF is non-specific, and a lack of healthcare worker awareness, that occurs in both endemic settings and in exported cases can result in delayed diagnosis of CCHF. This can delay initiation of supportive treatment for CCHF and also the required infection prevention and control measures. In our series there was a delay in recognition of CCHF in pregnant women in 4/8 cases (cases 3, 4, 5 & 6), all associated with severe/fatal disease and fetal death.

There is currently no specific antiviral therapy for CCHF, and the benefit of ribavirin treatment is controversial with mixed results mainly generated from retrospective studies.^20^ Its use is contraindicated in pregnancy (FDA Pregnancy Category X) due to significant teratogenecity being demonstrated in all animal species in which adequate studies have been performed.^21^ However, in ribavirin’s submission for inclusion in the WHO model list of essential medicines, its risk/benefit in pregnancy, in the context of the high mortality in VHF was re-considered.^22^ This was though predominantly based on its clearer evidence base in the treatment of Lassa fever and Argentinian Hemorrhagic fever^23^ and early reports of benefit in CCHF.

Ribavirin may have greater effect in late stage pregnancy where mortality appears to be higher, risk of teratogenicity lower, and we believe its use may be justified in this scenario. In our combined data when treatment and outcome was known, 5/13 pregnant women died who received ribavirin, compared to 15/23 who died who did not receive ribavirin. The difference is not statistically significant and caution must be applied to any interpretation and conclusions on interventions from this retrospective analysis. Unfortunately, Favipiravir, the only other antiviral that has shown survival benefit in CCHF animal models,^24^ is also contraindicated in pregnancy due to teratogenicity and embryogenicity^25^ Pregnancy was also an exclusion criteria for its use in the JIKI Ebola clinical trial.^26^ Limited data exists on the risk of transmission through breast feeding in CCHF, with only 2 cases reported.^27^ In both cases RT-PCR was positive in the mother’s blood and negative in breastmilk, and the babies did not go on to develop CCHF. In our units breastfeeding is not recommended during the acute illness phase of CCHF, due to established risks of CCHF transmission to the baby. Samples of breastmilk would then be sent for RT-PCR as the patient recovered.

There is currently no CCHF vaccine that is licensed by the European Medicines Agency or US Food and Drug Administration. An inactivated mouse brain vaccine was developed by the former Soviet Union in the 1970s, and is still in use in Bulgaria. However, it has failed to show high neutralizing antibody levels,^28^ and lacks controlled data demonstrating protective efficacy. A number of promising CCHF vaccine candidates are under development,^29^, ^30^ and will soon proceed to phase 1 clinical trials. Pregnant women in endemic areas should be considered a high-risk CCHF group that requires particular consideration with respect to vaccine safety evaluation and during future roll-out strategies.

Limitations to the systematic review are the incomplete information provided in some case descriptions, particularly on gestation of pregnancy. The total numbers identified also probably represent a significant underestimate due to incomplete reporting of cases from endemic regions and the likelihood of undiagnosed mild CCHF pregnant cases. Due to its non-specific clinical presentation CCHF in pregnancy can be difficult to distinguish from other causes of undifferentiated febrile illness. This is exacerbated by a lack of laboratory capacity in endemic regions combined with a lack of awareness of CCHF by clinicians. Hemorrhagic signs start around the fourth day of illness and it is often only at this stage that CCHF first considered, or as we have reported when there are secondary nosocomial cases.

## Conclusions

The number of reported cases of CCHF in pregnant women is low, but this is an underestimate and the geographical range of CCHF is increasing, including within Europe. Clinicians must maintain a high index of suspicion and undertake risk assessments for CCHF in pregnant women with a fever, who reside in or who have a history of travel to CCHF endemic areas. Early recognition allows appropriate infection prevention and control precautions to be put in place, reducing the demonstrated risk of nosocomial transmission. In accordance with other viral hemorrhagic fevers, the mortality of CCHF in pregnant women appears to be increased, but as we have highlighted supportive care focused on blood product replacement and access to critical care interventions can result in positive outcomes in severe disease. Novel therapeutics are required to improve both maternal and fetal outcomes in CCHF, and as such pregnant woman should be included in future CCHF clinical trials whenever possible.

## Conflict of interest

The authors declare that they have no competing financial interests. The content is solely the responsibility of the authors.

## Funding source

No specific funding. TF is funded by the Wellcome Trust (104480/Z/14/Z) and the UK Ministry of Defence.

## Figures and Tables

**Table 1:** Characteristic of pregnant CCHF cases.

Authors	Country	Year (s)	No. cases	Diagnosis	Age	Gestation	Maternal outcome	Hemorr-hage	Fetal/Neo-natal outcome	Ribavirin	Secondary cases
Al-Tikriti SK et al.^31^	Iraq	1979	3	Viral culture/clinical	ND	ND	Died	Yes	Died	No	No
					ND	ND	Died	Yes	Died	No	No
					ND	ND	Survived	Yes	Died	No	No
Baljosevic. S et al.^32^	Former Yugoslavia	1986–1995	4	UK	29	32/40	Died	Yes	Died	ND	ND
					30	24/40	Died	Yes	Died	ND	ND
					39	38/40	Died	Yes	Died	ND	ND
					24	11/40	Survived	Yes	Died	ND	ND
Sharif-Mood B et al.^11^	Iran	2000–2005	6	PCR/Serology	19–38	ND	Survived	Yes	Died	Yes	No
				PCR/Serology		ND	Survived	Yes	Died	Yes	No
				PCR/Serology		ND	Survived	Yes	Died	Yes	No
				PCR/Serology		ND	Survived	Yes	Survived	Yes	No
				PCR/Serology		ND	Survived	Yes	Survived	Yes	No
				PCR/Serology		16/40	Died	Yes	Died	Yes	No
Nabeth P et al.^19^	Mauritania	2003	2	Clinical	30	ND	Died	Yes	Died	No	Yes (19)
				Clinical	ND	ND	ND	ND	ND	No	ND
Ergonul O et al.^9^	Turkey	2003–2008	3	Serology	40	38/40	Survived	Yes	Died	Yes	No
				PCR	20	19/40	Survived	Yes	Died	No	No
				PCR/Serology	28	28/40	Died	Yes	Died	No	No
Gozel MG et al.^10^	Turkey	2007–2011	5	PCR/Serology	35	8/40	Survived	Yes	Died	No	No
				PCR/Serology	30	18/40	Survived	No	Survived	No	No
				PCR	41	20/40	Survived	No	Survived	No	No
				PCR/Serology	19	21/40	Survived	No	Survived	No	No
				PCR/Serology	27	33/40	Survived	No	Survived	No	No
Oskooei HO et al.^33^	Iran	2008	1	Serology	UK	UK	Survived	No	Survived	Yes	No
Dizbay M et al.^34^	Turkey	2009	1	PCR/Serology	22	36/40	Survived	Yes	Survived	Yes	No
Naderi HR et al.^18^	Iran	2009	2	(PCR +ve secondary cases)	UK	ND	Died	Yes	Died	No	Yes (1)
					31	ND	Died	Yes	Died	No	Yes (4)
Mumdchiev-a H et al.^35^	Bulgaria	2009	1	PCR/Serology	UK	26/40	Survived	Yes	Survived	UK	No
Aydemir O et al.^36^	Turkey	2010	1	PCR	29	30/40	Survived	No	Survived	No	No
Pshenichnya N et al.^2^	Russia	2011	1	PCR	23	22/40	Died	Yes	Died	No	Yes (8)
Mardani M et al.^37^	Iran	2011	1	PCR/Serology	24	16/40	Survived	Yes	Survived	Yes	No
Duygu F et al.^38^	Turkey	2011	2	PCR	25	17/40	Survived	Yes	Survived	No	No
				PCR	22	20/40	Survived	No	Survived	No	No
Ünlüsoy-Aksu A et al.^39^	Turkey	2014	1	PCR	23	36/40	Survived	Yes	Survived	Yes	No
Pschenichnaya N et al.	**Russia**	**2002**	**8**	**PCR/Serology**	**20**	**16/40**	**Survived**	**No**	**Survived**	**No**	**No**

	**Russia**	****2003****		****Serology****	****19****	****38/40****	****Survived****	****No****	**Survived**	**Yes**	**No**
	****Russia****	****2004****		****PCR****	****17****	****30/40****	****Died****	****Yes****	**Died**	**No**	**No**
	****Russia****	****2005****		****Serology****	****24****	****17/40****	****Survived****	****Yes****	**Died**	**No**	**No**
	****Kazakhstan****	****2009****		****Immunohist****	****23****	****40/40****	****Died****	****Yes****	**Died**	**No**	**Yes (5)**
	****Kazakhstan****	****2010****		****Immunohist****	****21****	****34/40****	****Died****	****Yes****	**Died**	**No**	**Yes (1)**
	****Russia****	****2011****		****PCR****	****17****	****18/40****	****Survived****	****No****	**Survived**	**Yes**	**No**
	****Turkey****	****2016****		****PCR****	****20****	****04/40****	****Survived****	****Yes****	**Died**	**No**	**No**

ND – Not determined. PCR – polymerase chain reaction, Immunohist – Immunohistochemistry.
